# Analysis of RXR/THR and RXR/PPARG2 Heterodimerization by Bioluminescence Resonance Energy Transfer (BRET)

**DOI:** 10.1371/journal.pone.0084569

**Published:** 2013-12-31

**Authors:** Miquel Mulero, Julie Perroy, Carole Federici, Gérard Cabello, Vincent Ollendorff

**Affiliations:** 1 INRA, UMR866 Dynamique Musculaire et Métabolisme, Montpellier, France; 2 Université Montpellier 1, Montpellier, France; 3 Nutrigenomics Research Group, Department of Biochemistry and Biotechnology, Rovira i Virgili University, Tarragona, Spain; 4 CNRS, UMR-5203, Institut de Génomique Fonctionnelle, Montpellier, France; 5 INSERM, U661, Montpellier, France; 6 Universités de Montpellier 1 & 2, UMR-5203, Montpellier, France; University of Geneva, Switzerland

## Abstract

**Background:**

Nuclear receptors (NR) regulate transcription of genes involved in many biological processes such as development, cell proliferation, differentiation and cell death. Amongst them, PPARG2 and THR control tissue glucose and lipid homeostasis which are deregulated in severe pathophysiological conditions such as metabolic syndromes.

**Methodology/Principal Findings:**

Here, we describe a real time BRET approach to monitor heterodimerization between RXR and PPARG2 or THR *in vitro* or in living cells. The presence of a specific DNA target was required to induce *in vitro* a BRET shift reflecting heterodimerization of RXR/PPARG2 or RXR/THR. As in electrophoretic mobility shift assay (EMSA), the stringency and specificity of the BRET shift assay depended upon assay condition optimization including MgCl_2_ concentration. For the nuclear receptors, we found by mutagenesis analysis that each heterodimer partner must harbor an intact DNA binding domain to induce BRET and heterodimerization on a DNA target. Moreover the interaction between the PPARG2 ligand binding domain and the RXR DNA binding domain stabilized the heterodimer on its DNA target. BRET microscopy in living cells highlighted the heterodimerization of RXR/PPARG2 within the nucleus clustered in discrete foci that may represent active target gene transcription regulation regions. BRET imaging also suggested that heterodimerization between RXR and PPARG2 required the DNA binding of PPARG2.

**Conclusions/Significance:**

The BRET approach described here allowed us to study the dynamic interactions which exist between NR *in vitro* or in living cells and can provide important information on heterodimerization modes, affinity with a given RE and subcellular localization of the heterodimers. This method could be used to study real time changes of NR heterodimers occurring on DNA depending upon cell activation, chromatin state and help to define the mechanisms of ligands or drug action designed to target NRs.

## Introduction

Nuclear receptors (NR) are members of a superfamily of ligand-activated transcription factors acting as transcriptional switches involved in the regulation of development, reproduction, and metabolism of lipids, drugs and energy. Genetic studies in humans and rodents support the notion that NRs control a wide variety of metabolic processes by regulating the expression of genes encoding key enzymes, transporters and other proteins involved in metabolic homeostasis [1]. The importance of this family of proteins in metabolic diseases is well supported by the use of NR ligands for the treatment of diabetes mellitus, dyslipidemia, hypercholesterolemia, or other metabolic disorders [2]. Amongst NRs, PPAR-gamma (PPARG) has been implicated in the pathology of numerous diseases including obesity, diabetes, atherosclerosis, and cancer. Interestingly, PPARG agonists have been used in the treatment of dyslipidemia and hyperglycemia [3], [4] and many insulin sensitizing drugs targetting PPARG are designed in the treatment of diabetes as a way to lower serum glucose without increasing pancreatic insulin secretion [5].

Most NRs share a modular structure characterized by a ligand-independent AF-1 transactivation domain in the N-terminus region, a highly conserved DNA binding domain including two zinc fingers recognizing specific DNA sequences called hormone response elements (HRE), and a ligand-binding and dimerization domain containing a ligand dependent AF-2 transactivation domain in its C-terminal portion [6]. For members of the subgroup II NRs, a typical HRE consists of two hexa-nucleotide motifs AGGTCA or its variants, separated by a gap of 1 to several nucleotides. Binding specificity by various receptors is largely achieved by the spacing (the 3–4–5 rule) and the orientation of both half-sites (direct, inverted or everted repeat).

Although a subset of them can bind and stimulate transcription as monomers or homodimers, NRs are more generally active as heterodimers and the retinoid × receptor (RXR) represents their main heterodimer partner [7]. NRs such as PPARG and thyroid hormone receptor (THR) are localized in the nucleus where they heterodimerize with RXR and bind to HREs. Two dimerization domains appear to work sequentially implying a to a two-step hypothesis for the binding of heterodimers to DNA. According to this model, the LBD (Ligand binding Domain) dimerization interface initiates the formation of solution heterodimers that, in turn, acquire the capacity to bind to a number of differently organized repeats. However, formation of a second dimer interface within the DNA binding domain restricts receptors to bind to DRs [8].

Consequently, heterodimerization has a three-fold effect: it leads to a novel response element repertoire, increases the efficiency of DNA binding relative to the corresponding homodimers, and allows two signaling inputs, that of the ligands of RXR and its partner. Crystal structures of DBD homo- and heterodimers have defined the surfaces involved in dimerization [9], [10]. It is important to point out that the response element repertoire for receptor homo- and heterodimers is dictated by the DBD while LBDs stabilizes the dimers, but do not contribute to response element selection.

In the absence of ligands, NRs interact with corepressor proteins inhibiting the transcription of the target gene. Binding of ligands results in a conformational change of the NR heterodimer that releases corepressor proteins allowing binding of coactivators. Other proteins of the preinitiation complex are then recruited and transcription by RNA polymerase II is activated [11].

Several methodologies have been developed to study the interaction between NRs and response elements (RE) and to monitor the effect of ligands. For example electrophoretic mobility shift assay (EMSA) is the reference method generally used to study *in vitro* binding of NRs on DNA RE and cell based reporter genes assays are often used to evaluate their activity and ligand responses [12]. Isothermal titration calorimetry is another *in vitro* method used to define in thermodynamic terms the magnitude and binding affinity between NRs and DNA RE. The main limitation of this last technique is the need to use purified proteins [13]. Additionally, the chromatin immunoprecipitation (ChIP) assay is a powerful and versatile technique used for probing protein-DNA interactions within the natural chromatin context; however this method requires formaldehyde fixation [14].

New approaches allowing dynamic studies are nevertheless required to study the NR interaction and function in living cells. Fluorescence redistribution after photobeaching and fluorescence resonance energy transfer (FRET) have been indeed used to show nuclear mobility of some NR in real time [15], [16]. The bioluminescence resonance energy transfer (BRET) cell based assay can also shed new light on the spatio-temporal dynamic of protein interactions in living cells, thus providing a method to focus on dynamic events occuring between NR following ligand binding or DNA recognition [17].

In this paper we describe a BRET approach to monitor RXR/PPARG2 and RXR/THR heterodimerization. BRET analysis in vitro and BRET imaging in living cells indicated that NR heterodimerization is strongly induced and stabilized by DNA binding. This method could be used to study real time changes affecting NR heterodimers depending on cell activation, or chromatin state and help to define how ligands or drugs designed to target NRs could affect their activities.

## Results and Discussion

The BRET method is based on resonance energy transfer between a light-emitting luciferase and a fluorescent acceptor. To develop a BRET interaction assay between RXR and PPARG2 or RXR and THR, we cloned their cDNAs in fusion with *Renilla luciferase* (Rluc8) and *enhanced yellow fluorescent protein* (EYFP) to obtain RXR-Luc, PPARG2-EYFP and THR-EYFP. Preliminary data demonstrated that higher BRET was observed by fusing Rluc8 or EYFP at the COOH-terminus of RXR, PPARG2 or THR proteins (data not shown) and only these donor and acceptor NR protein fusions were further characterized.

### NR Protein Fusions Required for BRET Localized in the Nucleus, Bound Consensus DNA RE and Activated Transcription

**Figure 1:**
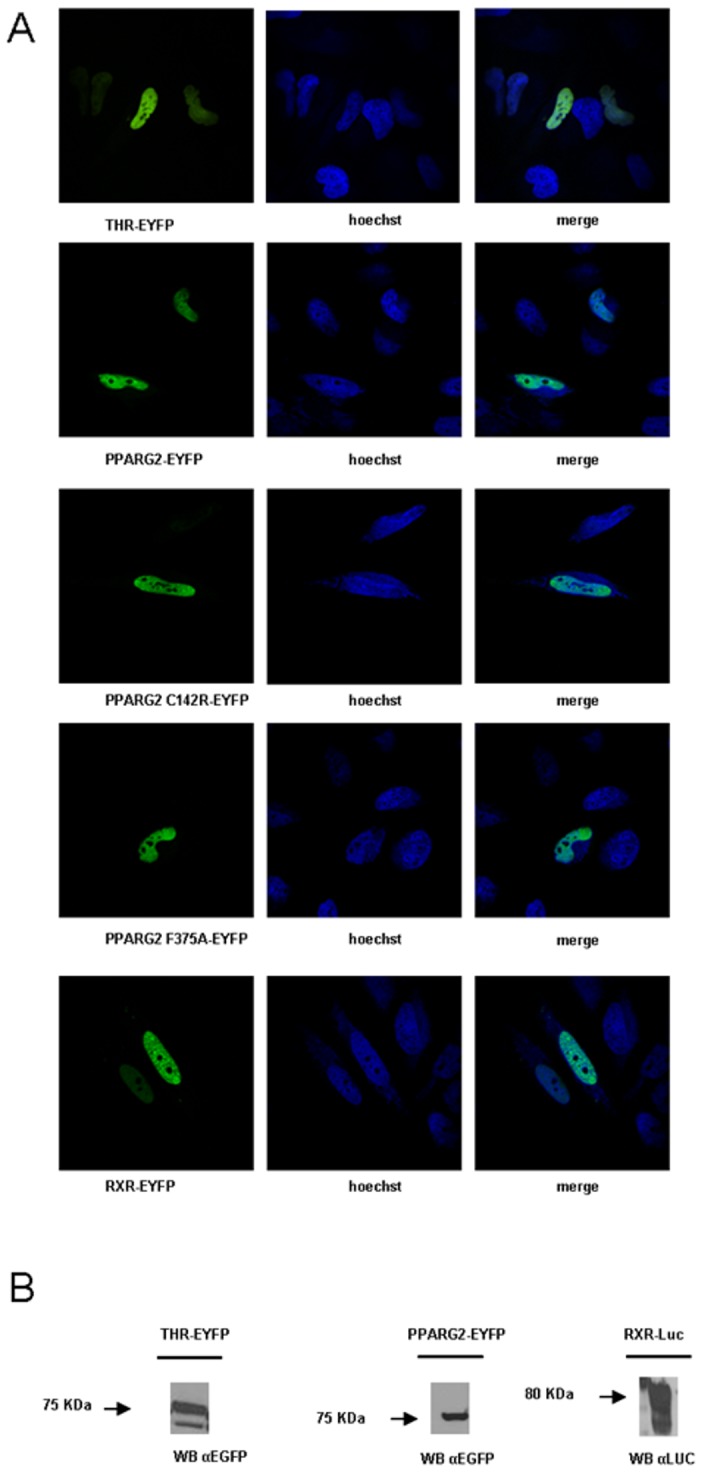
Expression and localization of NR fusion proteins. (A) Localization of THR, RXR and PPARG2 proteins in the nucleus. Hela cells transfected with the respective EYFP fused NR expression vector were stained with Hoechst 33342. Left images show the specific localization of each EYFP fusion protein; middle images show the nuclei revealed by Hoechst staining; right images show merge images between EYFP and DNA staining. (B) Protein identity confirmed by Western blot. HEK293T cells transfected with the respective EYFP or Luc fused NR expression vectors were lysed in RIPA buffer and analyzed by Western blot. Anti-GFP antibodies were used to detect THR-EYFP and PPARG2-EYFP, and anti-Renilla Luciferase antibody was used to visualize RXR-Luc.

**Figure 2:**
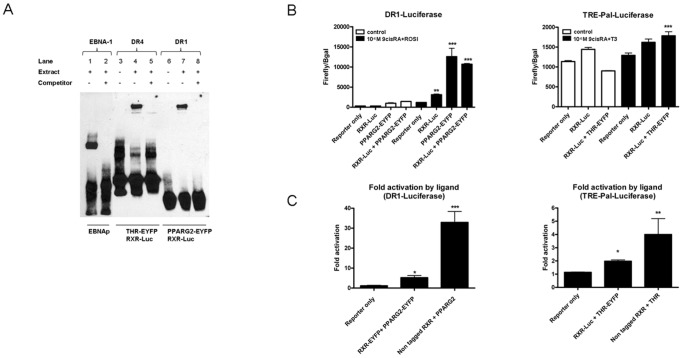
Functional validation of NR fusion proteins. (A) Electrophoretic mobility shift assay (EMSA) experiments with NR fusion proteins encoding RXR-Luc, THR-EYFP and PPARG2-EYFP. Nuclear extracts were prepared from HEK293T cells co-transfected with RXR-Luc and THR-EYFP or RXR-Luc and PPARG2-EYFP encoding plasmids. Several biotinylated RE oligonucleotides were mixed with nuclear extracts containing overexpressed NR fusion proteins: A DR4 probe was used to test the DNA binding of RXR-Luc+THR-EYFP (lane 4), and a DR1 probe was used for RXR-Luc+PPARG2-EYFP (lane 7). In the absence of nuclear extracts no signal was obtained (lanes 3 and 6). As a positive control for EMSA, a biotin-labeled 60 bp duplex bearing the Epstein-Barr Nuclear antigen 1 (EBNA-1) binding sequence was incubated with an extract containing the EBNA protein (lane 1). For each condition, the specificity of the gel shift experiment was determined by the addition of a 200-fold excess of the corresponding unlabeled double stranded consensus sequence (lanes 2, 5 and 8). (B) Histograms represent transcriptional activity of NR fusion proteins RXR-Luc, PPARG2-EYFP (left) or RXR-Luc and THR-EYFP (right) monitored with gene reporters containing DR1 responsive elements or thyroid responsive element palindomic (TREPAL) in their promoters: Hela cells were transiently transfected with RXR-Luc and PPARG2-EYFP or RXR-Luc and THR-EYFP expression plasmids together with a PPAR DR1 (left) or a TREPAL *firefly luciferase* gene reporter construct (right), respectively. A pCMV-β-galactosidase plasmid was used for normalization. 6 hours after transfection some cells were stimulated 24 h with 10^−6^ M rosiglitazone (ROSI) or 10^−6^ M of triodothyronine (T3) and 10^−6^ M of 9cis retinoic acid (9cis RA). After cells lysis, transcriptional activity was measured by monitoring firefly luciferase activities normalized by β-galactosidase activities. (C) Histograms represent transcriptional fold activation by ligand of transfected tagged constructs, RXR-Luc and PPARG2-EYFP (left) or RXR-Luc and THR-EYFP (right) compared to non tagged plasmids. Values shown are mean ± SD. (n = 2–3). Statistical differences were analyzed by one-way ANOVA followed by Bonferroni’s post hoc test: *P<0.05; **P<0.01; ***P<0.001.

As expected, these fusion proteins were localized in the nucleus of transfected cells (Fig. 1A) and we verified by western blot their correct molecular mass (Fig. 1B). Their DNA binding capabilities were also assessed by EMSA using consensus target sites. RXR-Luc in combination with PPARG2-EYFP or THR-EYFP could efficiently bind a biotinylated double strand consensus sequence direct repeat 1 (DR1) and direct repeat 4 (DR4) respectively (Fig. 2A).

Finally, the ability of these NR constructs to activate transcription was verified in gene reporter assays following transfection in Hela cells and an overnight (O/N) agonist stimulation with 10^−6^ M 9cis retinoic acid (9cisRA) and Rosiglitazone (ROSI) for RXR/PPARG2 and with 10^−6^ M 9cisRA and triiodotyronine (T3) for RXR/THR (Fig. 2B).

As expected, transfection of RXR-Luc and PPARG2-EYFP plasmids either alone or in combination, activated the DR1 responsive reporter gene and ligand incubation stimulated further gene reporter activity (Fig. 2B).

To test the transcriptional activity of THR-EYFP in combination with RXR-Luc we used a thyroid response element palindromic (TREPAL) gene reporter assay. As expected [18], [19], cells transfected with THR-EYFP and RXR-Luc inhibited the basal TREPAL reporter gene transcription whereas ligand incubation (9cisRA+T3) induced a significant transcriptional activation in cells cotransfected with THR-EYFP and RXR-Luc (Fig. 2B).

Compared to untagged versions, the fusion proteins RXR-Luc, PPARG2-EYFP and THR-EYFP exhibited a reduced ligand induced transcriptional activity (Fig. 2C).

Together this set of experiments demonstrated that the fusion proteins required for BRET studies behaved normally with respect to their subcellular localization and their ability to bind a consensus DNA RE and were able to activate transcription of a gene reporter.

### Heterodimerization of RXR/PPARG2 and RXR/THR Detected by BRET in Living Cells

**Figure 3:**
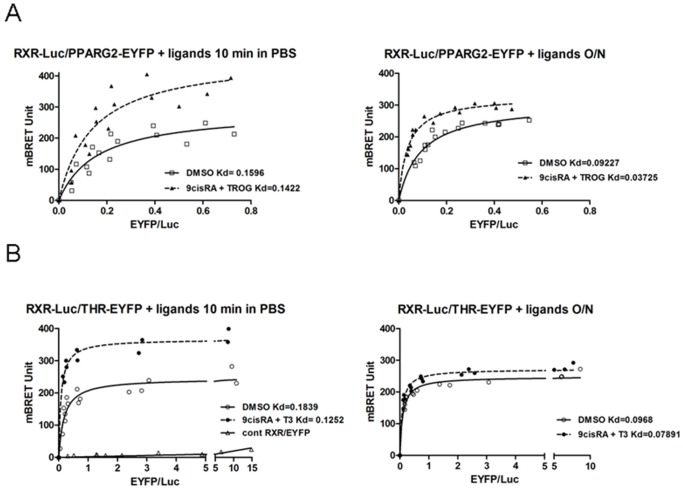
Titration BRET experiments in living cells. BRET between RXR-Luc and PPARG2-EYFP, RXR-Luc and THR-EYFP. Regression curves are represented with the BRET value as a function of the fluorescence/luminescence ratio (EYFP/Luc). HEK293T cells were transfected with a fixed amount of RXR-Luc donor plasmid together with increasing amount of acceptor plasmids (PPARG2-EYFP above or THR-EYFP and EYFP lower) and BRET was measured in living cells after stimulation with control DMSO or ligands either on resuspended cells 5–10 minutes in PBS (left panel), or on adherent cells during an overnight (O/N) incubation in culture medium (right panel). Values represent BRET measures (each in triplicate) integrated over a 20 min reading (A), BRET titration curves between RXR-Luc and PPARG2-EYFP from control cells (open square) and from cells stimulated 5 min with ligands 9cis RA+TROG (filled triangle) in PBS resuspended cells (left panel) or from adherent cells stimulated O/N (right panel) (B), BRET titration curves between RXR-Luc and THR-EYFP from control cells (open circles) and from cells stimulated 5 min with 9cisRA+T3 (filled circles) in PBS (left panel) or from adherent cells stimulated O/N (right panel) (right panel). A negative control saturation experiment is also shown between a RXR-Luc donor and an unfused EYFP acceptor protein (open triangle). Apparent affinity (apparent Kd) between RXR-Luc and PPARG2-EYFP or RXR-Luc and THR-EYFP represents EYFP/Luc ratio corresponding to the BRETmax/2 value (BRET50). Shown are cumulative data from three independent experiments in triplicate.

BRET experiments were then carried out following overexpression in HEK293T cells of donor and acceptor proteins. Under condition of constant level of RXR-Luc expression, BRET signal increased hyperbolically as a function of the PPARG2-EYFP (Fig. 3A) or THR-EYFP expression level (Fig. 3B), indicating a specific interaction between RXR and PPARG2 or THR [20]. A negative control saturation experiment between RXR-Luc donor and an unfused EYFP acceptor protein produced only bystander BRET signal due to random collision and confirmed the specificity of the BRET profiles observed between RXR-Luc and PPARG2 or RXR-Luc and THR-EYFP (Fig. 3B).

A short incubation of cells with agonists 2.10^−6^ M 9cisRA and 10^−5^ M of Troglitazone (TROG) for 5–10 min induced an increase of BRET max between RXR-Luc and PPARG2-EYFP. The increase in BRET signal likely attested to a conformational change of the activated receptors upon agonist binding. The protein level that was required to reach the BRET_50_ value (50% of the RXR-Luc linked to the PPARG2-EYFP) was not significantly changed in the presence of agonists (apparent Kd unchanged) (Fig. 3A), indicating that the affinity of the interaction between RXR and PPARG2 was largely independent of agonist binding. A similar conformational change was shown for RXR/THR heterodimerization following a short incubation with 2.10^−6^ M 9cisRA and 2.10^−7^ M of T3, without any significant change in the apparent affinity (Fig. 3B). This increase in BRETmax was more pronounced when cells were stimulated in PBS immediately before recording BRET (Fig. 3) compared to a 10 min ligand stimulation on adherent cells (Fig. S1). This can be explained by the experimental delay of 30 min required to prepare adherent cells before starting BRET monitoring (Fig. S1). After an O/N incubation of ligands, the increase of BRET signal was less pronounced than following a short time stimulation for RXR/PPARG2 and RXR/THR indicating that major conformational changes of NRs heterodimers occurred rapidly upon agonist binding and that these effects could be regulated over time. However, we noticed that the apparent Kd decreased for RXR/PPARG2 after an O/N ligand stimulation suggesting an increase of affinity between RXR and PPARG2. It is likely that an alteration of interaction of the RXR/PPARG2 heterodimer with DNA and/or with cofactors, or the chromatin state could be involved in this change of affinity between RXR and PPARG2 after O/N ligand stimulation.

### NRs Heterodimerisation is Induced by a Consensus DNA RE in vitro

An *in vitro* BRET assay was also set up to detect and reconstitute heterodimerization of NRs and to identify some parameters affecting their molecular interaction. HEK293T cells were transfected by either donor or acceptor NR fusion encoding plasmid. After 48 h, an *in vitro* BRET assay was performed by mixing different amounts of cell lysate containing an acceptor protein (PPARG2-EYFP or THR-EYFP) with a fixed amount of cell lysate containing the donor protein (RXR-Luc) with or without a double strand DNA (dsDNA) RE.

**Figure 4:**
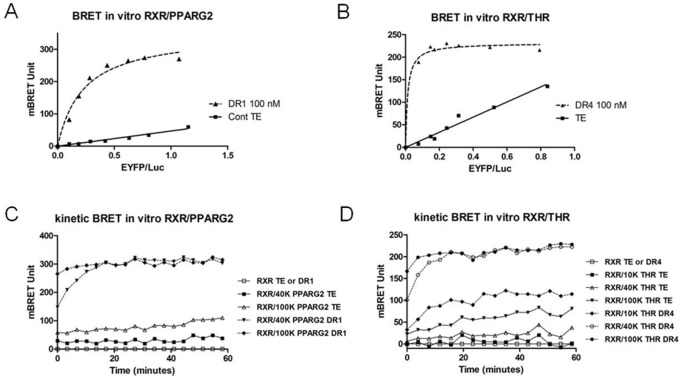
*In vitro* BRET shift between NRs in cleared cell lysates in the presence of a DNA RE. (A and B) For these *in vitro* titration BRET experiments, a fixed amount of PLB cell lysate expressing RXR-Luc protein (80 ku luciferase) is mixed with increasing amount of a PLB cell lysate expressing PPARG2-EYFP or THR-EYFP (0, 10, 20, 30, 50, 75, 100 or 150 ku of fluorescence) and regression curves are represented as the BRET value (recorded over a 20 min period) as a function of the fluorescence/luminescence ratio in the absence (control TE: Tris 10 mM PH 7,5; EDTA 1 mM) or presence of 100 nM of dsDNA RE (diluted in TE). (A) *In vitro* BRET saturation between RXR-Luc and PPARG2-EYFP with TE (control TE, filled squares) or 100 nM of dsDNA DR1 (filled triangles). (B) *In vitro* BRET saturation between RXR-Luc and THR-EYFP with TE (control TE, filled squares) or 100 nM of dsDNA DR4 (filled triangles). (C) One hour BRET kinetic monitoring *in vitro* interaction between 80 ku of donor RXR-Luc and 40 ku or 100 ku fluorescence of PPARG2-EYFP in the absence (control TE) or presence of 100 nM of dsDNA RE DR1. (D) One hour BRET kinetic monitoring *in vitro* interaction between 80 ku of donor RXR-Luc and 10 ku, 40 ku or 100 ku fluo of THR-EYFP in the absence (control TE) or presence of 100 nM of dsDNA RE DR4.

Analysis of the *in vitro* BRET saturation curves obtained, showed that the level of basal BRET was weak and increased linearly with the increase in fluorescence/luminescence (EYFP/Luc) ratio, most likely reflecting random collision between NRs and/or unstable heterodimerization (Fig. 4A and 4B). Interestingly, adding a pre-annealled consensus dsDNA RE induced an efficient heterodimerization between NRs (Fig. 4A and 4B). Indeed we observed a strong increase of BRET immediately following addition of a DR1 RE for RXR/PPARG2 and of a DR4 RE for RXR/THR (Fig. 4A and 4B). BRET max *in vitro* was reached with an acceptor/donor ratio corresponding to a minimum of 40 to 50 ku of fluorescence of acceptor protein for 80 ku of luciferase activity of donor protein in presence of 100 nM of consensus DNA binding target (DR1 or DR4). Further increasing the amount of acceptor did not greatly change BRET max but induced a higher basal BRET observed with no DNA RE (Fig. 4A and 4B). Analysing the BRET kinetics profiles of RXR/PPARG2 and RXR/THR showed that increasing the acceptor/donor ratio accelerated heterodimerization formation after addition of the DNA RE without changing the BRET max value achieved (Fig. 4C and 4D). However, the BRET max plateau eventually reached the same level 20 min after starting BRET recording (Fig. 4C and 4D compared 40 ku and 100 ku profiles).

To show that the DNA RE was required for BRET induction *in vitro*, we determined by BRET the heterodimerization of RXR/PPARG2 and RXR/THR over a 24 h period (Fig. S2). This experiment demonstrated that the presence of a DNA RE was absolutely required to induce heterodimerization and that no increase of BRET could be observed without it. Moreover, this showed that BRET and heterodimerization rapidly induced by a DNA RE, remained stable at least 24 hours at room temperature (Fig. S2).

### DNA RE Dose Response in BRET Shift Assay

**Figure 5:**
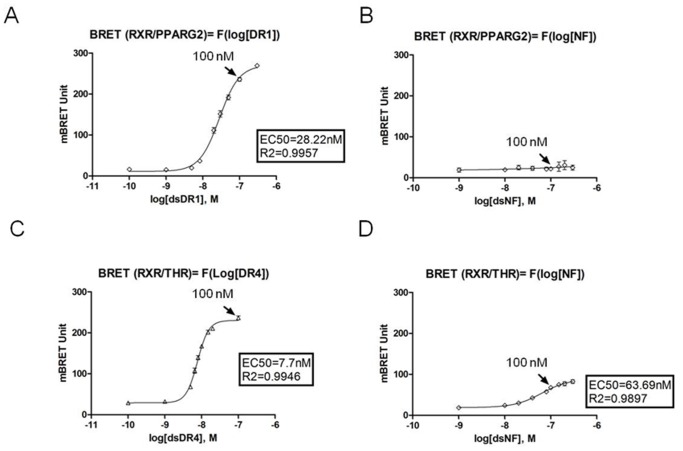
DNA dose responses of *in vitro* BRET shift. Dose response experiments showing *in vitro* BRET shift induced by different concentrations of a dsDNA RE. Values represent BRET measures (each in triplicate) integrated over a 10 min reading for DR1 and DR4 DNA RE and over a 20 min recording for NF DNA RE. (A), BRET values between RXR-Luc and PPARG2-EYFP as a function of the log[dsDNA] of DR1 (0,1 to 300 nM) (B), BRET values between RXR-Luc and PPARG2-EYFP as a function of the log[dsDNA] of NF (1 to 300 nM) (C) BRET values between RXR-Luc and THR-EYFP as a function of the log[dsDNA] of DR4 (0.1 to 100 nM) (D) BRET values between RXR-Luc and THR-EYFP as a function of the log[dsDNA] of NF. In each graph, the black arrow indicated the BRET value corresponding to a concentration of 100 nM of DNA RE. Shown are data from two to three independent experiments in triplicate and values represent mean ± SD. For each dose response, the R^2^ of the fitting slope and EC50 (corresponding to the half maximum effective concentration of a DNA RE) are shown.

We performed *in vitro* dose responses to determine the concentration of dsDNA consensus RE (DR1 or DR4) required to induce a maximal BRET response for RXR/PPARG2 and RXR/THR heterodimers (Fig. 5A and 5C). *In vitro* BRET dose responses with a negative control RE (NF for NfkB RE) induced no BRET response (from 1 to 300 nM) for RXR/PPARG2 heterodimer and a poor BRET induction only at high concentration for RXR/THR (Fig. 5B and 5D). The concentration of 100 nM of DNA RE was chosen since it was non limiting and sufficient to induce optimal BRET shift responses for a consensus RE.

Altogether these results established that a consensus DNA RE greatly induced and stabilized RXR/PPARG2 and RXR/THR heterodimers and modified their conformation allowing BRET and suggested that the increase of BRET monitoring heterodimerization could also reveal DNA binding.

### Analysis of DNA Binding Mutants in the BRET Shift Assay

**Figure 6:**
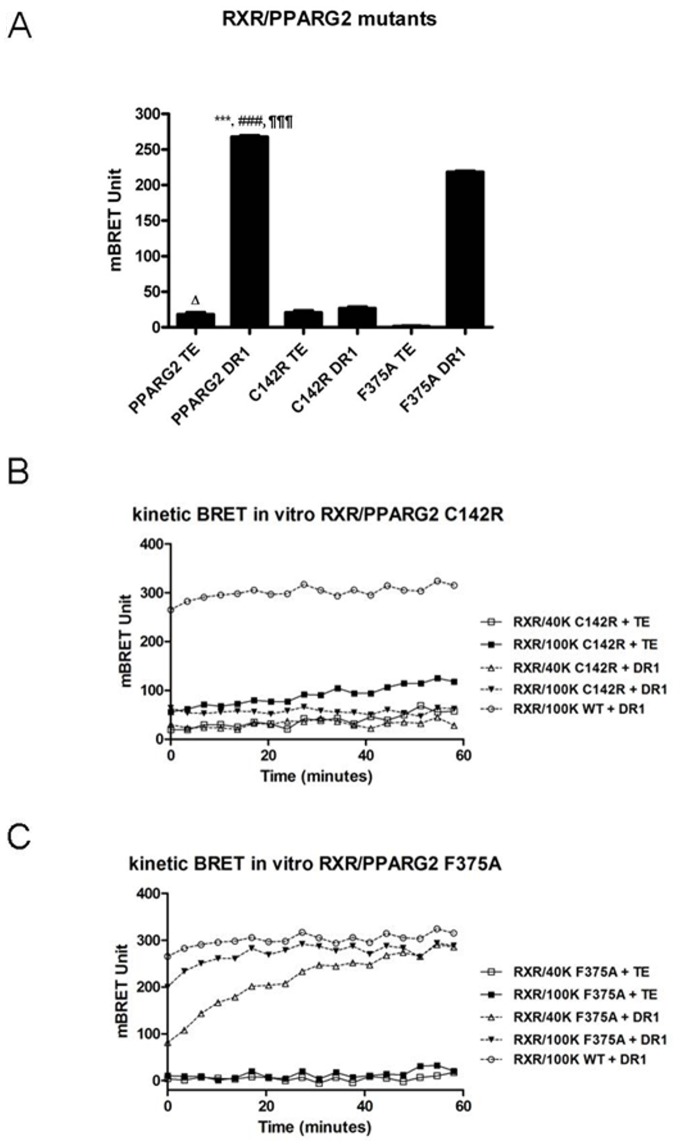
Analysis of DNA binding mutants. (A), BRET shift between 80 ku of RXR-Luc and 40 ku of PPARG2-EYFP-WT or mutants PPARG2-EYFP-C142R and PPARG2-EYFP-F375A in presence or absence of 100 nM DR1 dsDNA RE. Values represent BRET measures (each in triplicate) integrated over a 10 min reading. Values shown are means ± SD of three independent experiments (n = 3). Statistical differences of PPARG2WT DR1 relative to TE control (***P<0.001), PPARG2-C142R DR1 (^###^P<0.001) and PPARG2-F375A DR1 (^¶¶¶^P<0.001), as well as PPARG2WT TE control relative to PPARG2-F375A TE (^Δ^P<0.05) were analyzed by one-way ANOVA followed by Dunnett’s multiple comparison post hoc test. (B), One hour BRET kinetic monitoring interaction between 80 ku of donor RXR-Luc and 40 ku or 100 ku fluo of PPARG2-EYFPC142R in the absence (control TE) or presence of 100 nM of dsDNA RE DR1. (C), One hour BRET kinetic monitoring interaction between 80 ku of donor RXR-Luc and 40 ku or 100 ku fluo of PPARG2-EYFPF375A in the absence (TE) or presence of 100 nM of dsDNA RE DR1. For comparison with PPARG2WT in graphs 6B and 6C, a BRET kinetic recording interaction between 80 ku of donor RXR-Luc and 100 ku fluo of PPARG2-EYFPWT in the presence of 100 nM of dsDNA RE DR1 is shown (open circle).

To demonstrate that heterodimerization and BRET induced by a DNA RE depended upon DNA binding, we tested the effect of specific mutations known to abolish or destabilize the NR heterodimer interaction on its DNA target. For instance the DNA binding domain mutant PPARG2 C114R (corresponding to C142R in our PPARG2 isoform) is known to prevent any interaction with a DNA RE by altering its zinc finger domain and this mutation has been found in patients presenting a severe lipodystrophy [21]. Using this PPARG2 C142R mutant instead of PPARG2 WT as an acceptor abolished any BRET increase with RXR as a donor partner in presence of the DR1 consensus RE (Fig. 6A and 6B). Similarly, a mutant of RXR deleted for its DNA binding domain prevented any *in vitro* BRET shift and binding with PPARG2WT, PPARG2 mutants or THR on DR1 targets or DR4 respectively (Fig. S3 and data not shown). Moreover, deleting the DNA binding domain of THR abrogated its heterodimerization with RXR in presence of a DNA RE (data not shown). Altogether these data confirm that the BRET shift observed with wild type proteins is due to a physical binding of the heterodimer with the DNA target. As shown by other, these results illustrated also that each partner must harbor an intact DNA binding domain to get an efficient DNA binding of the heterodimer.

### The PPARG2 F375A Mutation Delayed Heterodimerization with RXR without Preventing DNA Binding

Based on structural features of the RXR/PPARG2 heterodimer bound to a consensus DR1 sequence target, Chandra et al. (2008) have discovered some important amino acid positions stabilizing the heterodimer interaction on DNA such as position F347 (corresponding to F375 in our PPARG2 isoform) in the ligand binding domain of PPARG [22]. According to Chandra et al. (2008) this residue stabilized the heterodimer on DNA by a hydrophobic interaction with the RXR DNA binding domain since a single F347A mutation of this residue prevents DNA binding [22].

In agreement with these published data, addition of the DR1 DNA target to RXR donor and PPARG2 F375A acceptor induced a BRET shift lower than the one observed with RXR and the PPARG2WT (p<0.0001) showing that the F375A mutation destabilized the heterodimer binding on DNA. However, we established here by the BRET shift method that the F375A mutation did not prevent heterodimerization and DNA binding as proposed by Chandra et al. (2008) based on EMSA experiments (Fig. 6A). Like for PPARG2 WT protein, the BRET between the PPARG2 F375A mutant and RXR was increased immediately after addition of a DNA RE (Fig. 6C kinetic F375A). However the time to reach the BRET max value depended upon the acceptor/donor ratio and was delayed for PPARG2 F375A compared to PPARG2WT (Fig. 6C). This illustrated the advantage of the BRET shift method in following the kinetic of heterodimerization and DNA binding in real time compared to a more static methods such as EMSA which is based on end point analysis. Moreover, PPARG2 and RXR proteins produced *in vitro* a low BRET observed without a DNA target that could reflect a basal heterodimerization (Fig. 6A). Interestingly, no such basal BRET was detected with PPARG2 F375A (Fig. 6A and 6C), suggesting that this mutation prevented any basal heterodimerization with RXR (Fig. 6C). Therefore the PPARG2 F375A mutation was not only able to destabilize or delay binding of the heterodimer on its DNA target but it could also affect heterodimerization formation with RXR free of DNA. Our data on this mutant extended previous published results and suggested that a precomplex formation between RXR and PPARG2 involving F375 could be an intermediate step to get an efficient binding of the heterodimer on its DNA target.

The analysis of the different DNA binding mutants demonstrated that the *in vitro* BRET shift method appeared appropriate to monitor in real time the interaction between NRs and a DNA target sequence.

### Stuctural Requirements of the DNA Targets

**Figure 7:**
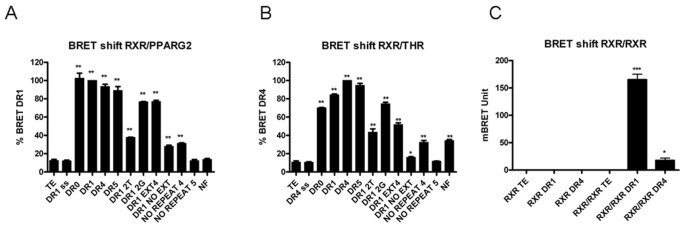
Stuctural requirements of the DNA targets. *In vitro* BRET shift recorded between RXR-Luc and EYFP-RXR, or RXR-Luc and PPARG2-EYFP, or RXR-Luc and THR-EYFP in the presence of 100 nM of different responsive elements (described in Table 1). Values represent BRET measures (each in triplicate) integrated over a 10 min reading. (A), BRET shift obtained between 80 ku of RXR-Luc and 40 ku of PPARG2-EYFP with control (TE), single strand (ss) consensus DR1 and 13 different double strand DNA RE. (B), BRET shift obtained between 80 ku of RXR-Luc and 40 ku of THR-EYFP with control (TE), single strand (ss) consensus DR4 and 13 different responsive elements. (C), BRET shift between 80 ku luciferase of donor RXR-Luc and 40 ku fluo of acceptor EYFP-RXR in absence (control TE) or in presence of 100 nM ds DNA DR1 or DR4 RE. Values shown are means ± SD (n = 3). Statistical differences relative to control (TE) were analyzed by one-way ANOVA followed by Dunnett’s multiple comparison post hoc test: ***P<0.001 and *P<0.05.

To study further the DNA RE specificity of the BRET response, several other DNA targets were also tested. (Fig. 7A, 7B and 7C, and Table 1).

**Table 1:** Different responsive elements used for *in vitro* BRET shift assays.

**DR0synth**	ATTTCTAGACT**AGGTCAAGGTCA**TCTAGACCC
**DR1synth**	ATTTCTAGACT**AGGTCA**A**AGGTCA**TCTAGACCC
**DR4synth**	ATTTCTAGACT**AGGTCA**CAGG**AGGTCA**TCTAGACCC
**DR5synth**	ATTTCTAGACT**AGGTCA**CCAGG**AGGTCA**TCTAGACCC
**DR1synth2T**	ATTTCTAGACT**TGGTCA**A**TGGTCA**TCTAGACCC
**DR1synth2G**	ATTTCTAGACT**AGGTCG**A**AGGTCG**TCTAGACCC
**DR1EXT4**	**AGGTCA** A**AGGTCA**G
**DR1NOEXT**	**AGGTCA** A**AGGTCA**
**NOREPEAT4**	CAAACT**AGGTCA**CATG
**NOREPEAT5**	CAAACT**AGGTCA**
**NF**	TGA**GGGGACTTTCC**CAGG

As expected single strand DNA did not induce any BRET shift demonstrating the specificity of the assay for dsDNA (Fig. 7A and 7B). Concerning RXR and PPARG2, heterodimerization was induced at maximum level by DR1 or DR0, whereas DR4 and DR5 induced also high BRET shift. Some discrete mutations of DR1 at the first position in both RE hemi-sites strongly altered the BRET response compared to a consensus DR1 RE (diminution of 60%) whereas mutating the 6xt positions induced only a small diminution of BRET shift. NF did not induce any BRET response and a DNA target containing no repeat induced poor or no BRET shift confirming that the repeated sequence of the RE is important for optimal BRET response and binding of the heterodimer.

As shown by others, we also observed that the size of the 5′ and 3′ extensions were important to stabilize NRs heterodimers on DNA. The heterodimerization was in fact significantly decreased with a DR1 consensus sequence having no 5′ extension and only one base remaining in the 3′ extension (DR1EXT4). Removing the last base in the 3′ extension (DR1NOEXT), abolished almost all BRET shift indicating that a perfect DR1 consensus core sequences with no base extension was not recognized anymore by RXR/PPARG2 heterodimers. These results are in agreement with 3D structural studies of the PPARG2/RXR complex on DNA that have established the importance of the 5′ and 3′ bases to stabilize the structure of the bound heterodimer on DNA [22].

The same kind of *in vitro* BRET analysis for RXR/THR heterodimer demonstrated a maximal induction of BRET shift for DR4 RE as expected. The BRET shift response remained high decreasing gradually with DR5, DR1 and DR0. As for RXR/PPARG2 heterodimer, the other DNA targets induced intermediate to low BRET shift responses (Fig. 7B). Homodimers RXR/RXR bind preferentially DR1 RE and we confirmed using the *in vitro* BRET assay that RXR homodimerization was strongly induced by DR1 but not by DR4 [23](Fig. 7C).

These results illustrated the different stuctural requirements of the DNA targets allowing efficient BRET (and binding) for RXR/PPARG2, RXR/THR or RXR/RXR dimers. It confirmed that binding and affinity of a heterodimer for a DNA target depends upon the composition of the core sequence, and the presence of surrounding bases. Concerning the spacing between heterosites, the small differences in BRET shift observed for RXR/PPARG2 and RXR/THR with DR0, DR1, DR4, and DR5 although significative, revealed a certain plasticity of binding not observed in gel shift by other authors. Therefore, we asked whether the specificity and intensity of the BRET shift responses could also depend upon assay conditions.

### Assay Conditions are Important for Heterodimerization and Binding Specificities

**Figure 8:**
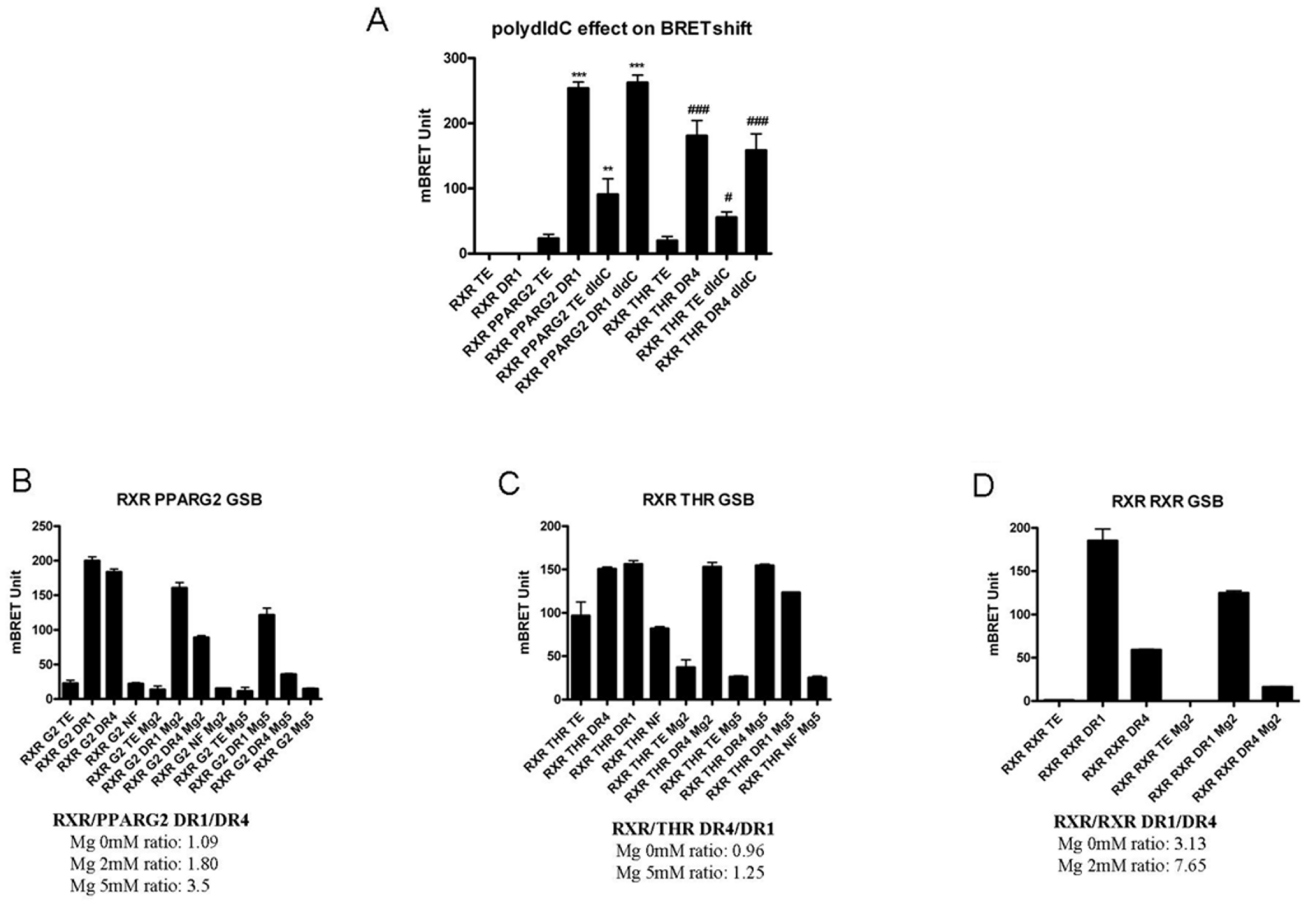
Effect of polydIdC and MgCl_2_ on *in vitro* BRET shift assay. (A) Effect of polydIdC addition on RXR/PPARG2 and RXR/THR BRET assay. A BRET shift assay in PLB was performed in presence or absence of polydIdC (50 ng/µl) and effect on basal BRET and specific DNA RE induced BRET shift was measured for RXR-Luc and PPARG2-EYFP (**P<0.01; ***P<0.001), or RXR-Luc and THR-EYFP (#P<0.05; ###P<0.001), Statistical differences relative to control (TE) were analyzed by one-way ANOVA followed by Dunnett’s multiple comparison post hoc test. (B, C and D) Effect of MgCl_2_ addition on BRET shift RXR/PPARG2, RXR/THR and RXR/RXR BRET assay. BRET shift experiments were carried out in Gel Shift Buffer (GSB) supplemented with 0, 2 or 5 mM of MgCl_2_. Calculation of the DR1/DR4 BRET shift ratio for RXR/PPARG2 (B), the DR4/DR1 BRET ratio for RXR/THR (C) and the DR1/DR4 BRET ratio for RXR/RXR (D), illustrated the higher specificity of BRET shift observed by increasing MgCl_2_ concentration. Histograms represent mean BRET value ± SD of at least 3 experiments in triplicate. Values represent BRET measures integrated over a 20 min reading.

In fact, as all *in vitro* DNA binding techniques, biochemical assay conditions can greatly affect the results (presence of polydIdC, concentration of MgCL_2_/salts/glycerol/NP40…). In EMSA experiments, the presence of a non specific DNA (polydIdC, salmon sperm DNA…) is for instance necessary to titrate out protein binding in a non specific way. We thus explored the effect of polydIdC in the BRET shift assay. In presence of 50 ng/µl of polydIdC, adding a specific dsDNA RE still induced a strong heterodimerization and BRET shift increase *in vitro* for RXR/PPARG2 and RXR/THR (Fig. 8A). However, we observed that “basal BRET” representing heterodimerization free of DNA RE, is slightly and significantly increased (Fig. 8A). It is possible that the “non specific” DNA environment of polydIdC could induce or stabilize a limited basal “preheterodimerization” state of NRs. Then, adding a specific DNA target will greatly favor an optimal recognition of the specific RE by the NRs, allowing heterodimerization and maximal BRET induction.

Our BRET assay was initially developed using a commercial cell lysis buffer that maintained high level of luciferase enzymatic activity, required for *in vitro* BRET measurements (PLB, Promega). We wanted to determine whether the BRET shift assay could also work in a well defined “home made” buffer such as the one used in a classical EMSA Gel shift experiment.

Initially, as seen in Figure 8 (panel B, C, D) heterodimerization remained nicely induced by addition of a consensus DNA RE in Gel shift Buffer (GSB) and the specificity of heterodimerization and DNA binding was increased in presence of MgCl_2_ as others had noticed for EMSA experiments [24]. It is believed that magnesium’s role is to inhibit non specific electrostatic interaction between a transcription factor and DNA [24]. It was indeed crucial to precisely set MgCl_2_ concentration for the BRET assay in GSB since it could modulate the relative binding of RXR/PPARG2 and RXR/THR for DR1 and DR4. Moreover MgCl_2_ helped to limit basal heterodimerization of RXR/THR (Fig. 8C).

Secondly, the low but significant BRET shift obtained for RXR/THR with a non specific DNA binding target (NF) in PLB was abolished in GSB showing how assay conditions can affect the result (see Fig. 7C and Fig. 8C).

Lastly, we found in GSB as in PLB the expected binding specificity of the RXR/RXR homodimer with a strong preference for a DR1 RE (Fig. 8D).

Therefore the biochemical conditions used classically in EMSA experiments also fit well to detect dimerization by BRET for RXR/PPARG2, RXR/THR or RXR/RXR in a specific DNA RE binding dependent manner.

In summary the “*in vitro*” BRET assay depicted in this study proved to be robust since it could be run in the same general assay conditions defined for EMSA experiments and allowed one to observe real time homo or heterodimerization of NRs and DNA binding. As in gel shift experiments, heterodimerization of NR and specificity of binding to a DNA target monitored by BRET depended upon some biochemical parameters requiring optimization. We observed particularly that MgCl_2_ could increase the specificity of DNA binding of RXR/THR and RXR/PPARG2 heterodimers.

### EMSA and *in vitro* BRET Method

EMSA is usually considered as a reference method to study heterodimer and transcription factor DNA binding *in vitro*. However some of the RE considered as poorly bound by a given NR heterodimer in EMSA (for instance the IGFBP-1 locus), can in fact constitute a very efficient binding and regulation site *in vivo* as determined by Chip [25]. Therefore it seems that EMSA results do not always reflect *in vivo* DNA binding. Moreover *in silico* research of PPARG consensus motif does not predict correctly *in vivo* binding of PPARG [26]–[28]. However analysis of large scale DNA chip studies have confirmed a certain degree of plasticity of binding showing on a genomic scale that endogenous PPARG2/RXR heterodimers are able to bind many different DNA sites spread out in the entire genome. Although the known PPARG/RXR binding motif is overrepresented in these binding regions these HRE sequences are sometimes far from a DR1 *stricto sensu* [27]. A certain flexibility in the binding of the primary core sequence appears therefore favorable to accomodate for sequence variability found in different responsive elements. This flexibility may reflect in part the physiological binding of a given NR heterodimer on different DNA targets with different affinities that could be translated in a fine tuning of gene target transcription [23]. For the *in vitro* BRET assay developed here, the DNA binding efficiency of the RXR/PPARG2 and RXR/THR heterodimers appeared to tolerate a certain plasticity in the primary sequence around the consensus DR sequence. However as for EMSA, we showed that assay conditions strongly influenced the specificity and stringency of binding and the level of BRET shift induced by a given DNA RE.

### Subcellular Localization of the Heterodimerization between RXR and PPARG2 WT or Mutants

**Figure 9:**
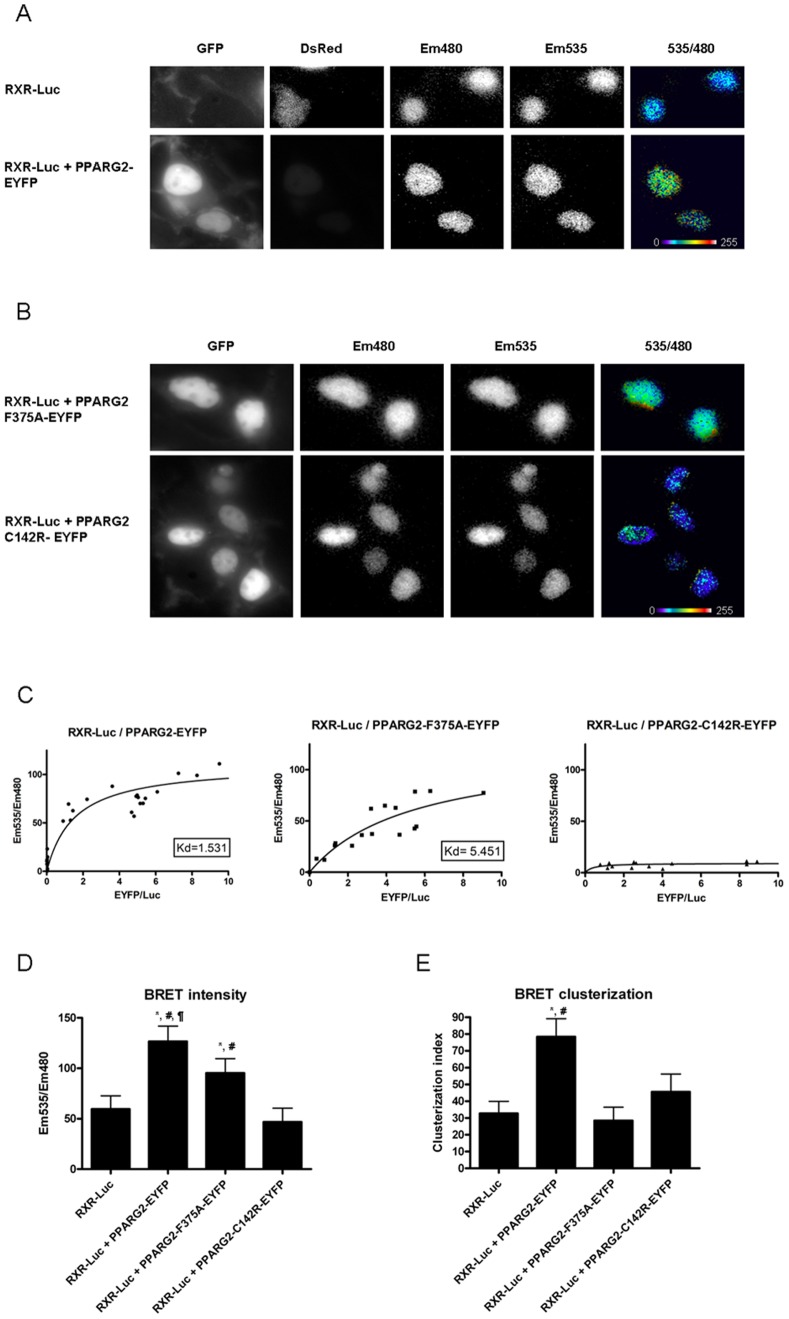
Subcellular localization of the interaction between RXR and PPARG2. BRET imaging was recorded in (A), HEK293T cells co-transfected with RXR-Luc and DsRed (as a transfection reporter) or RXR-Luc and PPARG2-EYFP or (B), HEK293T cells co-transfected with RXR-Luc and PPARG2-EYFP-F375A or PPARG2-EYFP-C142R. The pictures show expression of PPARG2-EYFP or mutants (GFP), RXR-Luc (Em480), PPARG2-EYFP or mutants excited by energy transfer (Em535) and BRET signal generated by the two tagged proteins (535/480). Please note the high and clustered BRET signals obtained between RXR-Luc and PPARG2-EYFP. The pixel-by-pixel 535 nm/480 nm ratios were calculated by dividing the absolute light intensities per pixel of images obtained at 535 nm over 480 nm. These numerical ratios (comprised between 0 and 1.5) were translated and visualized with a continuous 256 pseudo-color look-up table (LUT) as displayed in the figures (C) Titration curves were obtained by expressing for each cell the mean BRET intensity (Em535/Em480) as a function of the mean fluorescence/luminescence ratio (EYFP/Luc). Histograms represent the mean BRET intensity (D) and standard deviation (clusterization index) (E). A high standard deviation indicates a clusterization of the signal. Statistical differences were analyzed by one-way ANOVA followed by Bonferroni post hoc test: *P<0.05 (significant against RXR-Luc); ^#^P<0.05 (significant against PPARG2-EYFP-F375A);^ ¶^P<0.05 (significant against PPARG2-EYFP-C142R). The number of cells assayed for each condition was the following: 11 cells for RXR-Luc alone (control); 23 cells for RXR-Luc and PPARG2-EYFP; 15 cells for RXR-Luc and PPARG2-EYFP-F375A; and 21 cells for RXR-Luc and PPARG2-EYFP-C142R.

We then determined the subcellular localization of the interaction occurring between RXR and PPARG2 using BRET imaging in living cells [29]. Heterodimerization between RXR and PPARG2 took place within the nucleus (Fig. 9A). Moreover the BRET signal reflecting RXR/PPARG2 heterodimerization was confined to discrete foci (specific areas) as shown by calculation of a clusterization index (Fig. 9A and 9E). These foci may represent physiological interaction sites of RXR/PPARG2 heterodimers with specific DNA responsive elements involved in gene transcription regulation or preassembly zones. In fact other authors have shown nuclear redistribution of different PPAR isoforms by RXR at specific locations [30]. These nuclear foci have also been characterized for other NRs [16], [31] and van Royen et al. (2007) reported that compartmentalization of androgen receptor occurred in foci partly overlapping transcription sites.

Contrasting with the PPARG2WT protein, the PPARG2F375A mutant as a RXR partner produced a more uniform BRET signal throughout the nucleus (Fig. 9B, Fig. 9D and clusterization index Fig. 9E). This F375A mutation affecting heterodimer formation with RXR on DNA could reduce the amount of complex found on specific sites and eventually alter gene transcription regulation. Therefore the F375 residue of PPARG2 seemed important to localize the RXR/PPARG2 heterodimer at specific sites in the nuclei of living cells. Finally, using the PPARG2C142R mutant devoid of any *in vitro* DNA binding activity, no significant BRET was detected with RXR by BRET imaging, although both proteins were localized in the nucleus (Fig. 9B and Fig. 9D). Importantly, these low BRET values were obtained for similar range of Fluorescence (Acceptor)/Luminescence (Donor) ratios than for PPARG2F375A/RXR or PPARG2WT/RXR (see saturation curves Figure 9C) suggesting that no heterodimerization between RXR and PPARGC142R occur in living cells. Alternatively, the absence of BRET might be due to a bad orientation between the donor and acceptor entities, induced by the mutation. Nevertheless, as deduced from the *in vitro* assay, these real time BRET imaging studies with the PPARG2C142R mutant reinforced the concept that heterodimerization between RXR and PPARG2 was induced and stabilized by the DNA target and required an intact PPARG2 DNA binding domain. However, the existence of some basal heterodimer not bound with DNA can not be formally excluded. Indeed, using a mammalian two hybrid system, Agostini et al have observed an interaction between RXR and PPARG2 DNA binding mutants which depended upon their intact LBD interface [21]. This apparent discrepancy could come from differences in the nature and/or the sensitivity of the approaches employed.

Overall these results extended published datas showing that localization to specific nuclear foci depended upon heterodimerization between PPAR and RXR [30] and suggested that stable heterodimerization between RXR and PPARG2 required PPARG2 DNA binding.

### Dynamic Heterodimerization of RXR/PPARG2 and Binding on DNA RE

Several recent studies using large scale Chip sequence method have shown that natural DNA binding sites of endogenous RXR/PPARG exist by thousands and depend in part of the expression level of PPARG2 [32]–[34]. Consistently, in adipocytes and in macrophages most of the DNA sites found with RXR are also bound by PPARG2 showing that PPARG2 prefered RXR as a heterodimer partner to bind DNA [33], [35]. Moreover the presence of ligands is not required to detect by CHIP endogenous RXR/PPARG2 heterodimer or PPARG2 on most natural DNA binding sites [32] and Nielsen et al have established that endogenous RXR occupancy on DNA target sites is mainly dependent on PPARG [34]. Datas obtained during early differentiation of adipocytes demonstrated that even with a very low level of endogenous expression PPARG2 was found associated preferentially with RXR at targeted DNA sites [34]. This suggests that free RXR/PPARG2 heterodimer (not interacting with DNA) are probably rare and/or would only be transient states facilitating the formation of stable and abundant RXR/PPARG2 heterodimers bound on targeted DNA sites.

Accordingly our data suggest that the prefered heterodimerization form of RXR/PPARG2 is likely to be bound on specific DNA sites as PPARG2 DNA binding mutant failed to heterodimerize on DNA RE with RXR *in vitro* and did not heterodimerize with RXR in living cells. Therefore, endogenous RXR/PPARG2 heterodimers present in living cells are likely to be involved in a dynamic equilibrium between free and DNA bound heterodimer entities that will be interesting to characterize further.

Future experiments will be necessary to analyze the effect of other mutations on heterodimerization of RXR/PPARG2 and of different NRs to understand the dynamic events leading to heterodimer interaction and DNA recognition required for targeted gene transcription modulation.

### Conclusion

In summary, the *in vitro* and living cells BRET assays presented here can provide information on the interaction between NRs to gain insights about their mechanism of action. Our *in vitro* BRET assay showing heterodimerization of NRs on a DNA target appears as an interesting alternative method to EMSA to determine the spectrum of sequences possibly bound by NRs, to define the role of specific NR mutation and to follow the dynamic changes of these interactions. Moreover, the modulation in real time of NRs heterodimerization by agonists, antagonists or upon chromatin epigenetic modifications can also be investigated with this approach. The BRET imaging of NRs heterodimerization in living cells reported in this study, highlighted the role of PPARG2 in localizing RXR/PPARG2 in nuclear foci and suggested the importance of PPARG2 DNA binding to induce and stabilize a RXR/PPARG2 heterodimer.

The BRET appoaches described here should also help to screen for compounds modifying NR dependant gene regulation for the treatment of diseases such as metabolic disorders.

## Materials and Methods

### Electrophoretic Mobility Shift Assay (EMSA)

HEK293T cells were cotransfected with RXR-Luc and THR-EYFP or RXR-Luc and PPARG2-EYFP encoding plasmids. Cells were lysed with Passive Lysis Buffer (Promega, USA). Supernatants containing nuclear proteins were collected by centrifugation at 15 000 g for 30 min at 4°C and then stored at –70°C.

The corresponding nuclear extracts 5 µg were incubated with 20 fmol biotin labelled double stranded oligonucleotide containing: (a) the DR1 consensus sequence for the RXR/PPARG binding site or, (b) the DR4 consensus sequence for the RXR/THR binding site.

The retarded bands were detected by chemiluminescence using the LightShift Chemiluminescent EMSA kit (Pierce) following the manufacturer’s intructions. Briefly, biotin end-labeled duplex DNAs were incubated with the nuclear extract and electrophoresed on a native gel (6% nondenaturing polyacrylamide gel in 1X Tris-borate-EDTA buffer). The DNAs were then rapidly (30 minutes) transferred to a positive nylon membrane, UV crosslinked (10 minutes), probed with streptavidin-HRP conjugate and incubated with the enhanced chemiluminescence substrate. Digital images were taken with a GBOX Chemi XL 1.4 image system (Syngene). Specificity was determined by the addition of a 200-fold excess of unlabelled double stranded consensus sequences.

As a positive control, a biotin-labeled 60 bp duplex bearing the Epstein-Barr Nuclear antigen 1 (EBNA-1) binding sequence was incubated with an extract in which the EBNA-1 protein was overexpressed.

### Dual-luciferase Reporter Assay

Transcriptional activity of overexpressed RXR-Luc, PPARG2-EYFP and THR-EYFP in response to its ligands was assessed in Hela cells using a PPARG DR1 reporter assay (CCS-3026L, Qiagen, Netherlands) and a T3 responsive element palindromic (TREPAL) Luciferase plasmid containing 6 repeat of the pal0 motif AGGTCATGACCT upstream of a Thymidine Kinase promoter (a kind gift of Patrick balaguer, Montpellier). To correct for differences in transfection efficiency a β-galactosidase plasmid (pCMV-LacZ) was also co-transfected. Briefly, Hela cells were seeded into 12-well plates at a density of 50000 cells per well 1 day before transfection. The transfection was performed with the Attractene transfection reagent (Qiagen, Netherlands) according to the manufacturer’s guidelines. After 6 hours of transfection medium was replaced and cells were washed with PBS and treated with ligands (10^−6^ M 9cisRA and 10^−6^ M T3 or 10^−6^ M rosiglitazone). After 24 h, cells were lysed with Passive Lysis Buffer (PLB, Promega, USA). The mixtures were centrifugated at 10 000 g for 10 min at 4°C, and the supernatant was preserved at −80°C. Activity of firefly luciferase was measured in a luminometer Biotek FLx800 Multi-Detection Microplate Reader using the luciferase assay system (Promega, USA). β-Galactosidase activity was measured by using a mammalian β-galactosidase assay kit (Thermo Scientific, USA). Promoter activity was quantified by calculating for each sample the ratio of firefly luciferase activity/β-gal activity. All the luciferase assays were carried out at least in duplicate, and the experiments were repeated twice. Statistical analysis was carried out by one-way analysis of variance (ANOVA) with Bonferroni’s post-hoc test using Prism 4.00 (GraphPad Software, USA). Differences were considered significant when P<0.05.

### Western Blot Analysis

Cells were harvested and homogenized in RIPA lysis buffer (50 mM Tris-Cl pH 7.4, 150 mM NaCl, 1% NP40, 0.25% Na-deoxycholate, containing protease and phosphatase inhibitors). Aliquots of cell lysate containing 30 µg of protein per sample were analyzed by western blot. Membranes were then incubated overnight with primary monoclonal antibodies against Renilla Luciferase (Mab 4410 Millipore), or GFP (Roche). The blots were washed thoroughly in TBS-T buffer and incubated for 1 h with a peroxidase-conjugated IgG antibody. Immunoreactive proteins were visualized using an enhanced chemiluminescence substrate kit (ECL plus; Amersham Biosciences, GE Healthcare) according to the manufacturer’s instructions.

### Immunohistochemistry

Hela cells growing on glass coverslips were subjected to transient transfection with different EYFP fused NRs expressing plasmids using JETPEI or JETPrime transfection agent (Polyplus, France). 24 later, cells were washed with PBS and fixed for 30 min with a 4% paraformaldehyde solution in PBS. Cells were then washed in PBS and stained for 5 min with 1 mg/ml Hoechst in PBS for visualization of the nucleus. Coverslips were mounted with Prolong gold antifade reagent (Molecular Probes) and visualized with a Zeiss LSM510 confocal microscope.

### BRET Constructs

To make BRET vectors expressing donor and acceptor nuclear receptors, mouse RXRα, mouse THRα and human PPARG2 cDNAs were cloned in fusion with Rluc8 and EYFP plasmids using PCR and the Gateway technology (Invitrogen). Following are the name and accession number of the different NR cDNAs used in this study: Mouse RXRα, NP_035435, size 467aas (starting Methionine 1), mouse RXRα delta (deletion of the DNA binding domain), 265 aas (starting Methionine 203), mouse THRα, NP_835161 size 410aas (starting Methionine 1) and human PPARG2, NP_056953 size 505 aas (starting Methionine 1). We cloned Rluc8 and EYFP in N-terminus and C-terminus of RXR, THR and PPARG2. Preliminary experiments showed that the C-terminus fusion proteins gave more BRET signal and these fusions were therefore selected for all experiments described in this study.

The codon stop was removed and due to the cloning process (system Gateway, Invitrogen) the last aminoacid of each NR fusion is followed by a small common stretch of aminoacid (…N P A F L Y K…) that links to the donor (Rluc8) or the acceptor (EYFP) sequence.

Mutations to produce PPARG2 C142R and F375A were made using a mutagenesis kit following manufacturer instructions (Stratagene). All constructs were verified by sequencing.

In some experiments (reporter assays) untagged RXRα, THRα and PPARG2 plasmids were also used and a plasmid encoding unfused EYFP was used as an empty vector or for BRET saturation control experiments.

Oligonucleotides required to make all the constructs described in this paper are available upon request.

### BRET Analyses in Living Cells

BRET titration experiments were performed by fixing the amount of the donor RXR protein (fused to Rluc8 *Renilla Luciferase*) and by increasing the amount of the THR or PPARG2 acceptor protein fused to enhanced yellow fluorescent protein (EYFP) coexpressed by transient transfection in HEK293T cells. 300 000 cells distributed in 6 wells plates were transfected one day after plating using JET PEI (4 µl/µg of DNA, Polyplus) with a total of 1 µg DNA/well containing each 50 to 200 ng of a plasmid encoding RXR-Luc donor protein and increasing amount of the acceptor plasmid (0 ng, 50 ng, 100 ng, 200 ng, 400 ng or 600 ng). pBluescript was used to normalize DNA amount to 1 µg.

To measure BRET signal, 48 hours after transfection, cells were collected and resuspended in 300 µl of PBS containing 0,1% of glucose. Cells were deposed in triplicate in a white 96 wells microplate and Coelenterazine H (interchim) was added to each well in PBS (final concentration 5 µM). BRET signal was measured over a 10 to 20 minutes period with a Biotek synergy2 reader that allows to sequentially detect the emission signal at 530 and 480 nm (1 sec reading for each wavelength). The BRET signal was then calculated by determining the emission ratio 530/480 and by substracting the background 530/480 ratio of cells expressing only donor protein. Following Coelenterazine H hydrolysis, the donor protein fused to luciferase emits light in a spectrum range allowing excitation of the EYFP acceptor protein and BRET signal. To evaluate the level of each expressed donor protein, total luminescence was measured by calculating the mean of the triplicate initial reading at 480 nm immediately following Coelenterazine H addition. Similarly total fluorescence was measured to quantify the level of each expressed acceptor protein fused to fluorescent EYFP following excitation at 485 nm and reading at 530 nm.

For a titration experiment, the total BRET signal was plotted as a function of the total Fluorescence (EYFP)/Luminescence (Luc) ratio. A specific BRET interaction must increase hyperbolycally as a fonction of the acceptor/donor (Fluorescence/Luminescence) ratio while non specific interactions and random collisions would increase linearly. BRET saturation (BRETmax value) is reached when all expressed donor proteins are involved in an interaction with an acceptor protein. Affinity between RXR and THR or PPARG2 (apparent Kd) is the value of EYFP/Luc ratio corresponding to the BRETmax/2 value (BRET50). Experiments were performed 3 to 5 times.

### BRET Shift *in vitro*

Following 48 h hours transfection with donor or acceptor plasmids cells were washed in PBS and lysed in Passive Lysis Buffer (PLB, Promega, USA) and cleared cell lysates were obtained after centrifugation at 4°C at 12000 g during 10 mn. Amount of donor proteins were estimated by measuring the total Luciferase unit of the extract; similarly the amount of acceptor proteins was determined by measuring EYFP fluorescence. For a typical assay 80 000 (80 ku) light unit of donor LucRXR was mixed with 50000 (50 ku) fluorescence unit of acceptor protein (PPARG2-EYFP, THR-EYFP or RXR-EYFP) and completed to 40 µl with PLB. 10 µl of TE (Tris 10 mM PH 7,5; EDTA 1 mM) or ds DNA RE diluted in TE were added to the mix. BRET was then monitored over a 10 min to 60 min period by successive reading of one second at 480 nm and 530 nm immediately after addition of Coelenterazine H in PBS (5 µM final) in a microplate Biotek Synergy2 reader and the ratio 530/480 was calculated. Each condition was loaded in triplicate wells. Calculation of BRET was done as usual by substracting to each 530/480 ratio value the basal 530/480 ratio of control wells containing only donor protein.

Similar *in vitro* BRET shift assay was also performed by replacing the PLB by a Gel shift Buffer (GSB) containing: 10 mM tris PH 7,5/50 mM KCl/1 mM DTT supplemented with various concentration of MgCl_2_ (0/2 or 5 mM). Cleared concentrated cellular extracts in PLB containing known amount of donor or acceptor proteins were diluted in GSB and *in vitro* BRET shift assay was performed as explained above (GSB replacing PLB).

DNA responsive elements used in the BRET shift assay were prepared as followed: Oligonucleotides were synthesized by Invitrogen, and complementary strands were resuspended and mixed at 10 µM of dsDNA in Tris 10 mM PH 7,5; NaCl 150 mM and EDTA 1 mM. For annealing, mix of complementary oligonucleotides were boiled 2 min and left to cool off in a water bath over a 20–30 min period. The dsDNA RE obtained were then diluted to the working concentration in TE (Tris 10 mM PH 7,5; EDTA 1 mM). In some control experiments single strand (ss) DNA was used. In some experiment polydIdC (sigma) was added to the *in vitro* BRET assay.

### BRET Imaging

BRET imaging has been previously described [29], [36]. Briefly, all images were obtained using a bioluminescence-dedicated Axiovert 200 M inverted fluorescence microscope (Zeiss, Jena, Germany) with a Plan-Apochromat 63×/1.40 Oil M27 objective at room temperature. Transfected cells were first identified using a monochromatic light and an appropriated filter to excite EYFP (exciter HQ480/40 #44001– emitter HQ525/50 #42017, Chroma). The light source was then switched off until the end of the experiment. Coelenterazine H (CoelH, 20 µM) was applied 5 min before acquisition. Images were collected using an Evolve camera from Photometrics. Sequential acquisitions of 20 sec were performed at 5 MHZ – Gain 3950, binning 1, with emission filters D480/60 nm (#61274, Chroma) and HQ535/50 nm (#63944, Chroma) to select Em480 and Em535 wavelengths respectively, using the Metamorph software (Molecular Devices). The pixel-by-pixel 535 nm/480 nm ratios were calculated by dividing the absolute light intensities per pixel of images obtained at 535 nm over 480 nm. These numerical ratios (comprised between 0 and 1.5) were translated and visualized with a continuous 256 pseudo-color look-up table (LUT) as displayed in the figures. To determine the average intensity and distribution of the 535 nm/480 nm ratio, we calculated the mean intensity and standard deviation of pixels within a square region of the cell of interest using Image J software (NIH). To obtain the saturation curves, we quantified in each cell the expression level of the acceptor by measuring the mean fluorescence obtained by light excitation before addition of CoelH and the expression level of the donor by measuring the luminescence in presence of CoelH (Em480). The BRET ratio Em535/Em480 was then expressed as a function of the expression levels (Fluo/Lumi ratio).

## Supporting Information

Figure S1 **Titration BRET experiments in living cells.** BRET between RXR-Luc and PPARG2-EYFP, RXR-Luc and THR-EYFP. Regression curves are represented with the BRET value as a function of the fluorescence/luminescence ratio (EYFP/Luc). HEK293T cells were transfected with a fixed amount of donor plasmid (encoding RXR-Luc) together with increasing amount of acceptor plasmids (encoding PPARG2-EYFP above or THR-EYFP) and BRET was measured in living cells after stimulation with control DMSO or ligands 10 minutes in DMEM. Values represent BRET measures (each in triplicate) integrated over a 20 min reading (A), BRET titration curves between RXR-Luc and PPARG2-EYFP from control adherent cells (open square) and from adherent cells stimulated 10 minutes with 9cis RA+TROG in DMEM (filled triangle) (B), BRET titration curves between RXR-Luc and THR-EYFP from control adherent cells (open circles) and from adherent cells stimulated 10 minutes with 9cis RA+T3 (filled circles). Shown are cumulative data from three (LucRXR/PPARG2-EYFP) or two (RXR-Luc/THR-EYFP) independent experiments in triplicate.
(TIF)

Figure S2 **Stability of BRET and heterodimerization induced by a DNA RE** ***in vitro*** **.** Cells transfected 48 h by donor RXR-Luc or acceptor plasmid (PPARG2-EYFP or THR-EYFP) were lysed in PLB and donor and acceptor protein present in each centrifugated cleared cell lysate were quantified. *In vitro* BRET monitoring were performed on cleared lysates by mixing 80 ku of donor RXR-Luc with 50 ku fluo of acceptor PPARG2-EYFP or THR-EYFP in presence or in absence of 100 nM of DNA RE. Graphs show BRET changes recorded during a 24 h period after mixing. BRET was measured at different time points: immediately after mixing, and 30 min, 1 hour, 3 h, 6 h and 24 h after mixing. RXR-Luc and THR-EYFP with TE (open triangle) RXR-Luc and THR-EYFP supplemented with 100 nM DR4 (filled triangle), RXR-Luc and PPARG2-EYFP with TE (open square) RXR-RLuc and PPARG2-EYFP supplemented with 100 nM DR1 (filled square).
(TIF)

Figure S3 ***In vitro*** **BRET shift kinetics with LucRXR delta DNA binding domain.** Graphs represent one hour BRET kinetics monitoring interaction between 80 ku of donor RXR-Luc deleted of its DNA binding domain (deltaRXR-Luc) and 40 ku or 100 ku of EYFP- PPARG2 (top graph), PPARG2-EYFP C142R (middle graph) or PPARG2-EYFP F375A (lower graph) in the absence (control TE) or presence of 100 nM of dsDNA RE DR1.
(TIF)
