# Supplementary figures and images for: Analysis of RXR/THR and RXR/PPARG2 Heterodimerization by Bioluminescence Resonance Energy Transfer (BRET)
Source: PLoS One. 2013 Dec 31;8(12):e84569. doi: 10.1371/journal.pone.0084569 (PMC3877338; doi:10.1371/journal.pone.0084569)

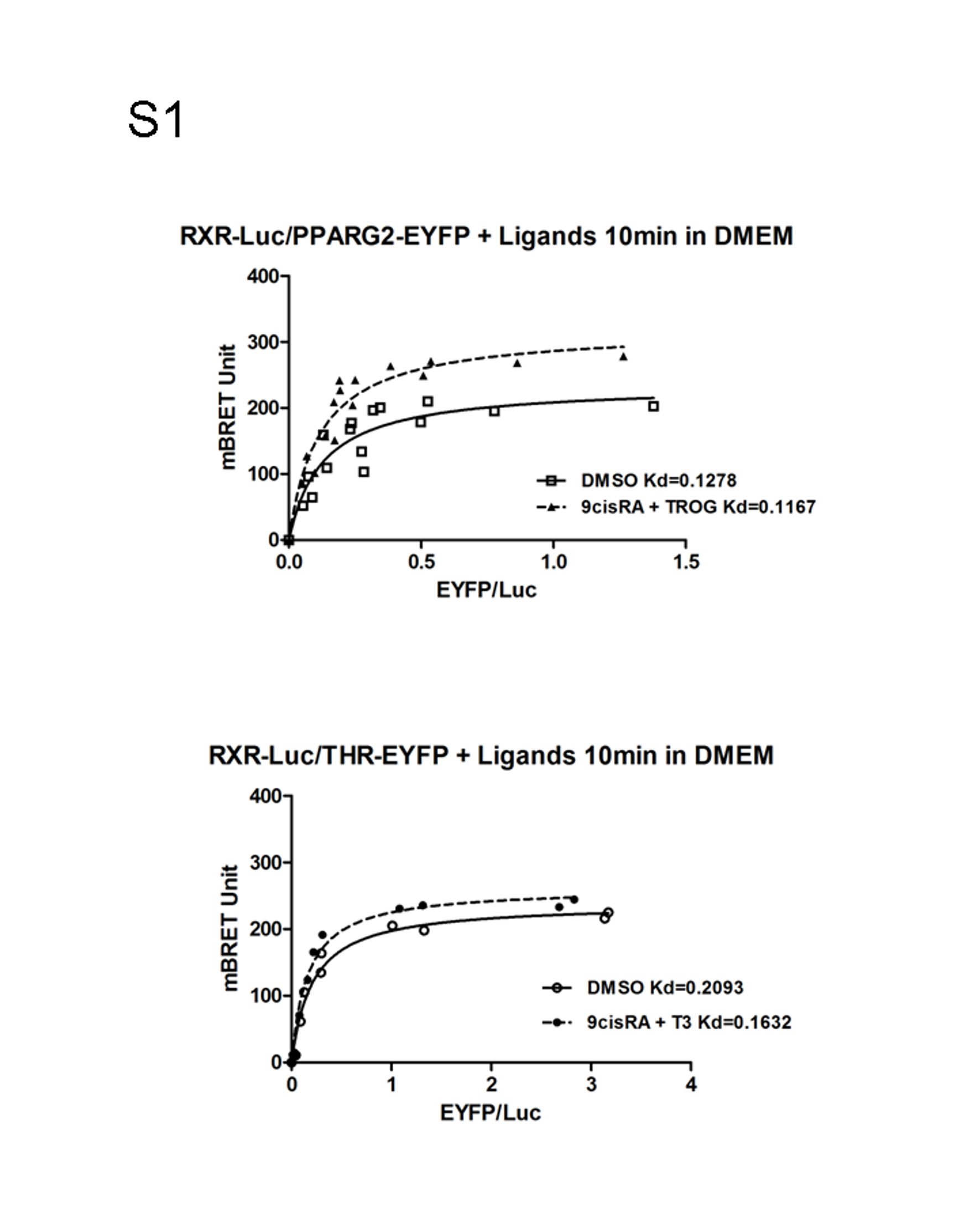

Supplement: Figure S1 — Titration BRET experiments in living cells. BRET between RXR-Luc and PPARG2-EYFP, RXR-Luc and THR-EYFP. Regression curves are represented with the BRET value as a function of the fluorescence/luminescence ratio (EYFP/Luc). HEK293T cells were transfected with a fixed amount of donor plasmid (encoding RXR-Luc) together with increasing amount of acceptor plasmids (encoding PPARG2-EYFP above or THR-EYFP) and BRET was measured in living cells after stimulation with control DMSO or ligands 10 minutes in DMEM. Values represent BRET measures (each in triplicate) integrated over a 20 min reading (A), BRET titration curves between RXR-Luc and PPARG2-EYFP from control adherent cells (open square) and from adherent cells stimulated 10 minutes with 9cis RA+TROG in DMEM (filled triangle) (B), BRET titration curves between RXR-Luc and THR-EYFP from control adherent cells (open circles) and from adherent cells stimulated 10 minutes with 9cis RA+T3 (filled circles). Shown are cumulative data from three (LucRXR/PPARG2-EYFP) or two (RXR-Luc/THR-EYFP) independent experiments in triplicate. (TIF) [file pone.0084569.s001.tif]

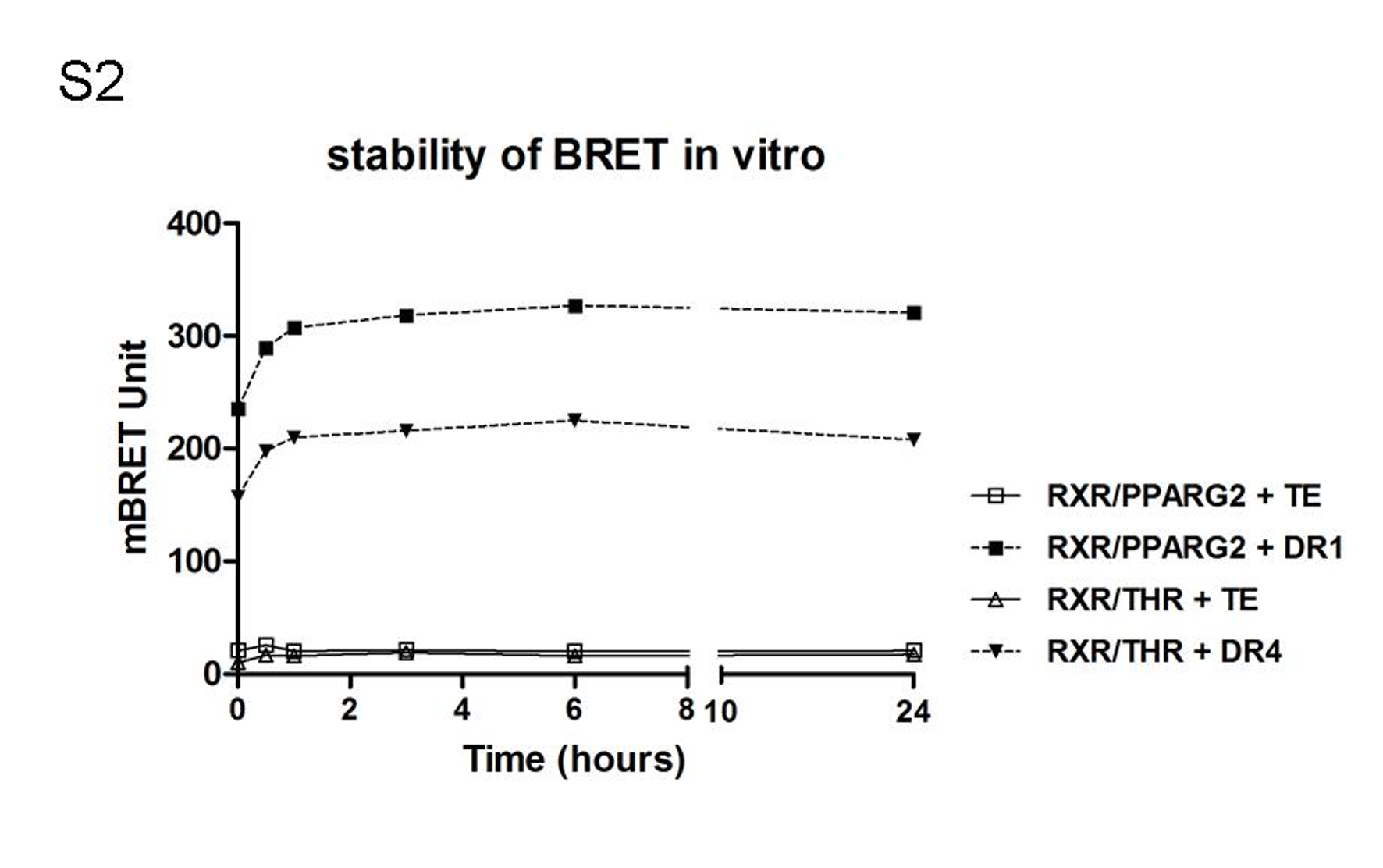

Supplement: Figure S2 — Stability of BRET and heterodimerization induced by a DNA RE in vitro . Cells transfected 48 h by donor RXR-Luc or acceptor plasmid (PPARG2-EYFP or THR-EYFP) were lysed in PLB and donor and acceptor protein present in each centrifugated cleared cell lysate were quantified. In vitro BRET monitoring were performed on cleared lysates by mixing 80 ku of donor RXR-Luc with 50 ku fluo of acceptor PPARG2-EYFP or THR-EYFP in presence or in absence of 100 nM of DNA RE. Graphs show BRET changes recorded during a 24 h period after mixing. BRET was measured at different time points: immediately after mixing, and 30 min, 1 hour, 3 h, 6 h and 24 h after mixing. RXR-Luc and THR-EYFP with TE (open triangle) RXR-Luc and THR-EYFP supplemented with 100 nM DR4 (filled triangle), RXR-Luc and PPARG2-EYFP with TE (open square) RXR-RLuc and PPARG2-EYFP supplemented with 100 nM DR1 (filled square). (TIF) [file pone.0084569.s002.tif]

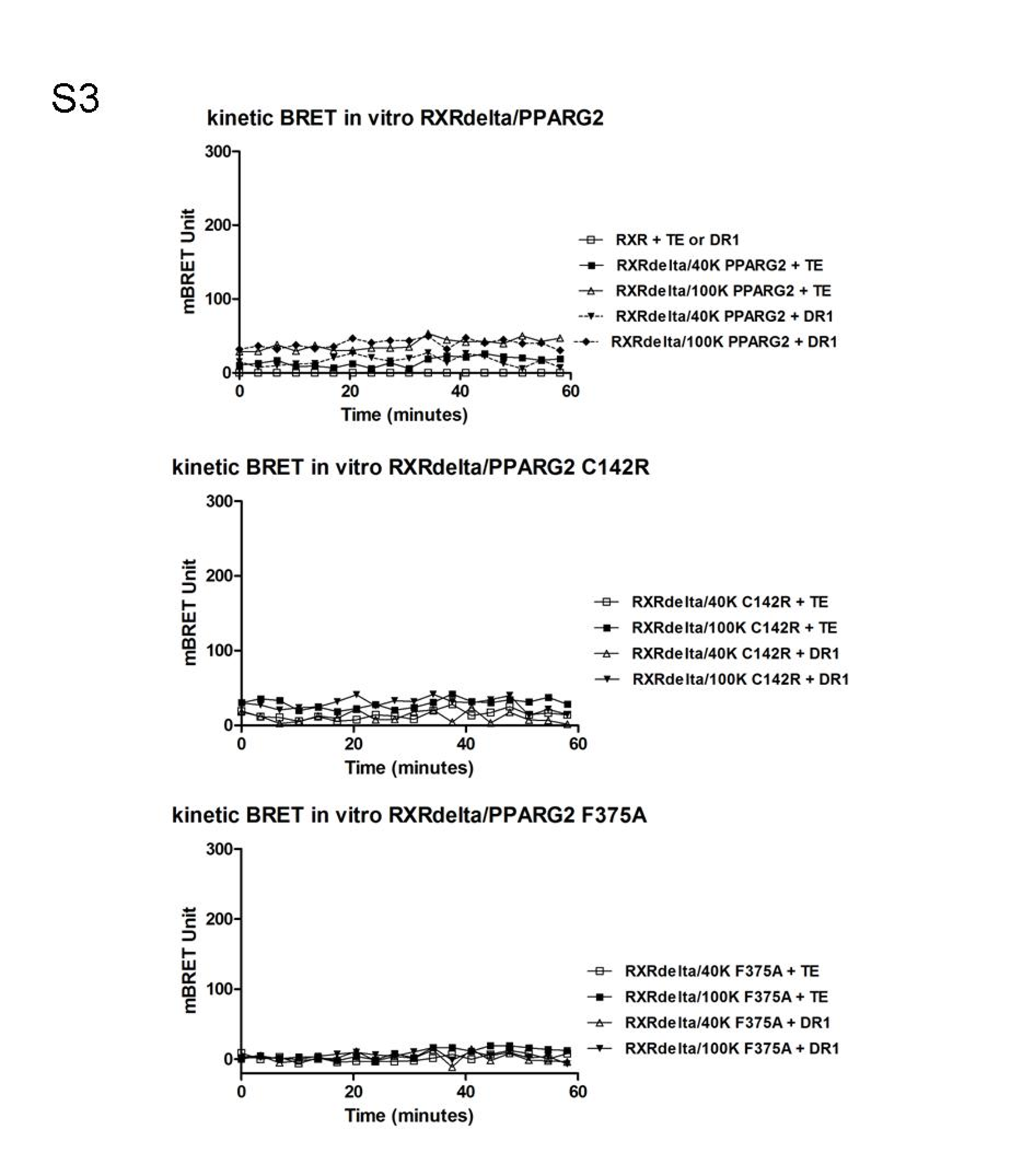

Supplement: Figure S3 — In vitro BRET shift kinetics with LucRXR delta DNA binding domain. Graphs represent one hour BRET kinetics monitoring interaction between 80 ku of donor RXR-Luc deleted of its DNA binding domain (deltaRXR-Luc) and 40 ku or 100 ku of EYFP- PPARG2 (top graph), PPARG2-EYFP C142R (middle graph) or PPARG2-EYFP F375A (lower graph) in the absence (control TE) or presence of 100 nM of dsDNA RE DR1. (TIF) [file pone.0084569.s003.tif]
